# Risk analysis index demonstrates superior predictive performance compared to traditional frailty metrics in elderly female patients undergoing inpatient total shoulder arthroplasty

**DOI:** 10.1186/s42836-025-00366-3

**Published:** 2026-03-05

**Authors:** Cameron J. Sabet, Bhav Jain, Bara M. Hammadeh, Abdulhalim Kikhia, Mohammad D. Alfawareh

**Affiliations:** 1 Georgetown University Medical Center, Washington, DC 20007 USA; 2 Stanford Medicine, Stanford, CA 94305 USA; 3 Faculty of Medicine, Al-Balqa’ Applied University, Salt, 19117 Jordan; 4 Tishreen University, Latakia, 2230 Syrian Arab Republic; 5 Department of Spine and Orthopedic Surgery, King Hussein Cancer Center, Amman, 11941 Jordan

**Keywords:** Risk Analysis Index, Frailty Assessment, Total Shoulder Arthroplasty, Surgical Outcomes, Geriatric Surgery

## Abstract

**Background:**

While frailty assessment has become integral to preoperative risk stratification, the optimal measurement tool remains unclear for elderly women undergoing total shoulder arthroplasty (TSA). This study compared the predictive performance of the Risk Analysis Index (RAI) against traditional metrics, including the modified frailty index-5 (mFI-5) and Geriatric Nutritional Risk Index (GNRI) in this specific population.

**Methods:**

We conducted a retrospective analysis of ACS NSQIP data from 2015–2021, including female patients aged 65–89 undergoing inpatient TSA. RAI incorporates age, functional status, recent weight loss, and physiological markers, including renal failure, congestive heart failure, and dyspnea. The mFI-5 assesses five comorbidities (diabetes, hypertension, COPD, heart failure, functional dependence), while the GNRI evaluates nutritional status using albumin and body weight. The discriminative ability of RAI, mFI-5, and GNRI was assessed using area under the curve (AUC) analysis for multiple 30-day outcomes. Primary outcomes were non-home discharge and extended length of stay (≥ 4 days), selected based on their clinical importance for discharge planning and quality metrics. Secondary outcomes included 30-day mortality, major and minor complications, readmission, and reoperation. Discriminative ability was assessed using area under the curve (AUC) analysis. Internal validation was performed using bootstrap resampling.

**Results:**

Among 11,965 patients analyzed, RAI demonstrated superior predictive performance for primary outcomes with AUCs of 0.784 for non-home discharge and 0.670 for extended length of stay, significantly outperforming mFI-5 (AUCs 0.601 and 0.590, respectively) and GNRI (AUCs 0.544 and 0.543). For secondary outcomes, RAI maintained competitive performance across mortality, complications, readmissions, and reoperations.

**Conclusion:**

The Risk Analysis Index provides superior discrimination for non-home discharge and extended length of stay compared to traditional frailty measures in elderly female TSA patients, with particularly strong predictive performance for discharge disposition, supporting its adoption as the preferred risk stratification tool for discharge planning in this population.

Video Abstract

**Supplementary Information:**

The online version contains supplementary material available at 10.1186/s42836-025-00366-3.

## Introduction

Total shoulder arthroplasty (TSA) represents one of the most rapidly expanding procedures in orthopedic surgery, with annual volumes exceeding 104,000 cases in the United States—a number projected to rise further due to increasing life expectancy and the growing prevalence of degenerative joint disease in older adults [1]. As surgical volumes increase, accurately predicting postoperative risk in elderly populations is an urgent clinical priority. Frailty, distinct from chronological age, has emerged as a dominant predictor of poor outcomes across a range of surgical procedures. In shoulder arthroplasty specifically, frailty has been linked to heightened risks of complications, readmission, reoperation, and non-home discharge [2, 3].

Several frailty indices have been proposed to aid perioperative risk stratification, most notably the modified frailty index (mFI), which has demonstrated predictive validity across hip, spine, and shoulder surgery cohorts [4, 5]. The mFI-5, a streamlined version incorporating five key comorbidities, has gained widespread adoption due to its simplicity and derivation from readily available clinical variables. Nutritional indices such as the Geriatric Nutritional Risk Index (GNRI) and functional dependency metrics have also shown independent prognostic significance [6, 7]. However, these measures are typically assessed in isolation, meaning that clinicians evaluate comorbidity burden, nutritional status, and functional capacity separately rather than integrating them into a single composite risk score, limiting their clinical utility in guiding comprehensive perioperative care.

Significant limitations persist with traditional frailty metrics. Recent studies have raised concerns about the declining discriminatory power of mFI-based tools in contemporary surgical datasets. Gani et al. [8] demonstrated that missing data in the NSQIP database significantly affected mFI calculation, with 100% of data missing for five of the eleven composite variables after 2012. Shultz et al. [9] showed that systematic changes in the NSQIP database resulted in inconsistent mFI calculations over time, potentially affecting the validity of studies utilizing this index. Additionally, Aziz et al. [10] found that the mismanagement of missing data in large clinical databases using mFI could lead to inaccurate findings.

The Risk Analysis Index (RAI) represents a more comprehensive frailty assessment tool, incorporating not only comorbidities but also functional status, recent weight loss, and physiological markers of decline. Originally developed and validated in general surgery populations, RAI has shown promise in orthopedic applications. Desai et al. [11] demonstrated that functional status—a key component of RAI—was particularly predictive of complications in upper extremity procedures. Phen et al. [12] found that incorporating functional assessment with nutritional parameters improved risk prediction in lower extremity fracture patients. The RAI’s emphasis on functional dependence and recent weight loss makes it particularly well-suited for elderly female TSA patients, in whom baseline functional capacity and nutritional decline are critical determinants of postoperative recovery and discharge disposition.

Prior literature has not adequately addressed the need for a unified, composite risk score that synthesizes multiple validated predictors into a single, interpretable model specifically for shoulder arthroplasty. This study addresses this critical knowledge gap by providing a comprehensive comparative analysis of frailty assessment tools in elderly female TSA patients. We hypothesized that the RAI would demonstrate superior predictive performance compared to mFI-5 and GNRI across key postoperative outcomes, offering clinicians an evidence-based framework for risk stratification in this growing surgical population.

## Material and methods

### Data source and study design

This retrospective cohort study utilized the American College of Surgeons National Surgical Quality Improvement Program (ACS NSQIP) Participant Use File from 2015 through 2021. NSQIP represents a prospectively collected, risk-adjusted, multi-institutional database capturing detailed perioperative data from over 700 participating hospitals across the United States. The database includes more than 200 variables encompassing preoperative risk factors, intraoperative variables, and 30-day postoperative outcomes. This study adhered to the Strengthening the Reporting of Observational Studies in Epidemiology (STROBE) guidelines for observational research. Given the de-identified nature of the publicly available NSQIP dataset, this study was exempt from institutional review board approval and informed consent requirements.

### Patient selection criteria

The study population included female patients aged 65 to 89 years who underwent inpatient total shoulder arthroplasty, identified using Current Procedural Terminology (CPT) code 23,472. Exclusion criteria were applied systematically to ensure data quality and cohort homogeneity: (1) patients aged 90 years or older due to NSQIP age top-coding limitations; (2) male patients; (3) presence of preoperative infection or disseminated malignancy (Preoperative infection was identified using the NSQIP variable for systemic sepsis, systemic inflammatory response syndrome, or septic shock present at the time of surgery. Disseminated malignancy was identified using the NSQIP variable indicating cancer that has spread to other sites or is considered incurable; (4) missing data for discharge destination, mortality status, or functional status; and (5) incomplete variables necessary for calculating frailty indices. After applying these criteria, 11,965 patients with complete data were included in the final analysis. American Society of Anesthesiologists (ASA) physical status classification was recorded as a descriptive variable characterizing overall perioperative risk, but was not included as a comparator frailty index given its lack of specificity for frailty assessment.

### Frailty index definitions

Risk Analysis Index (RAI): The RAI was calculated based on validated methodology incorporating multiple domains of frailty assessment. The clinical RAI (RAI-C), as described by Hall et al. [13] was utilized for this analysis. The full RAI typically includes cognitive impairment and cancer treatment status; their exclusion may result in underestimation of frailty burden in some patients. However, prior NSQIP-based RAI studies have demonstrated that the adapted index retains strong predictive validity despite these omissions. Variables included age, sex (all female in this cohort), recent weight loss (> 10% in 6 months), functional status (independent, partially dependent, or totally dependent), renal failure, congestive heart failure, dyspnea, and disseminated cancer. Due to NSQIP limitations, cognitive impairment and cancer treatment variables were excluded from the calculation. Patients were stratified into four categories: robust (RAI ≤ 20), prefrail (RAI 21–30), frail (RAI 31–40), and severely frail (RAI ≥ 41). Of note, the RAI does not include participation in sports or cultural activities as component variables; these factors are not part of the validated RAI instrument.

Modified Frailty Index-5 (mFI-5): The mFI-5 was calculated as the sum of five binary variables: diabetes mellitus, hypertension requiring medication, chronic obstructive pulmonary disease or recent pneumonia, congestive heart failure, and non-independent functional status. All five mFI-5 component variables were consistently available in the NSQIP dataset across the study period. Scores ranged from 0 to 5, with patients categorized as not frail (0), prefrail (1), frail (2), or severely frail (≥ 3). These categorization thresholds were derived from prior validation studies in orthopedic populations [4]. Geriatric Nutritional Risk Index (GNRI): The GNRI was calculated using the formula: GNRI = (1.489 × albumin g/L) + (41.7 × present weight/ideal body weight). Patients were stratified into four nutritional risk categories: no risk (GNRI > 98), low risk (GNRI 92–98), moderate risk (GNRI 82–91), and severe risk (GNRI < 82). Serum albumin and weight variables required for GNRI calculation were available for all patients in the final cohort; patients with missing albumin values were excluded during cohort selection. Risk stratification thresholds were based on the original GNRI validation by Bouillanne et al. [14].

### Outcome measures

Primary outcomes were pre-specified on clinical grounds as non-home discharge and extended length of stay (≥ 4 days), given their importance as quality metrics, surrogate markers for functional decline, and relevance to discharge planning in elderly surgical patients. An AUC of 0.700 or greater was interpreted as indicating good discriminative ability, consistent with established guidelines for evaluating diagnostic accuracy. These included non-home discharge and extended length of stay (≥ 4 days). Secondary outcomes encompassed 30-day mortality, major complications (myocardial infarction, cardiac arrest, stroke, pulmonary embolism, sepsis, organ space surgical site infection), minor complications (urinary tract infection, blood transfusion requirement, superficial surgical site infection), unplanned readmission, and unplanned reoperation.

### Statistical analysis

Descriptive statistics were calculated for all variables, with continuous data presented as means with standard deviations or medians with interquartile ranges based on distribution normality. Categorical variables were expressed as frequencies and percentages. Bivariate comparisons across frailty tiers utilized the Kruskal–Wallis test for continuous variables and chi-square tests for categorical variables.

The predictive performance of each frailty index was assessed using receiver operating characteristic (ROC) curve analysis, with area under the curve (AUC) values calculated for each outcome. AUC values were interpreted according to established guidelines: 0.5–0.6 indicating poor discrimination, 0.6–0.7 fair, 0.7–0.8 good, 0.8–0.9 excellent, and > 0.9 outstanding discriminative ability [15]. The DeLong method was employed to test for significant differences between AUC values of different indices. Multivariable logistic regression models were constructed to evaluate independent associations between frailty indices and outcomes, adjusting for relevant covariates.

Internal validation was performed using bootstrap resampling with 100 iterations to assess model stability and generate bias-corrected confidence intervals. All statistical analyses were conducted using Stata MP Version 18 within the Redivis computing environment. Statistical significance was defined as a two-sided *P*-value < 0.05. This validation approach was designed to assess discriminative ability and model stability rather than to establish a calibrated risk threshold; future studies incorporating external validation cohorts will be necessary to define clinical decision thresholds.

## Results

### Patient demographics and clinical characteristics

**Fig. 1:**
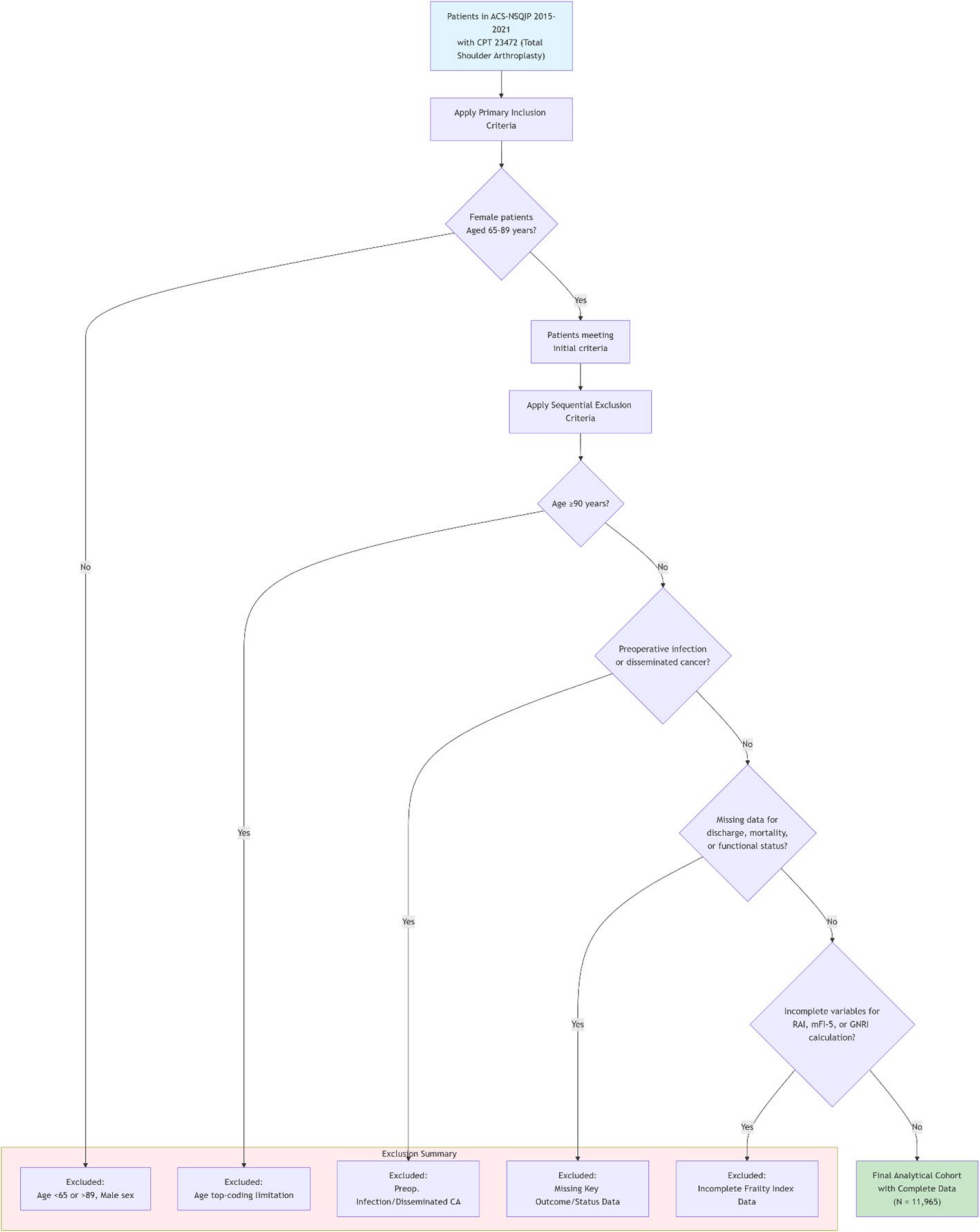
CONSORT flowchart of patient selection process

The final cohort comprised 11,965 female patients with a mean age of 70.5 ± 8.8 years and a mean body mass index of 31.6 ± 7.5 kg/m^2^. The majority were White (92.6%) and functionally independent at baseline (97.2%). Complete demographic and comorbidity data are presented in Table 1. Figure 1 displays the patient selection flow diagram. All 11,965 patients included in the final cohort had complete data for RAI, mFI-5, and GNRI calculation, ensuring that identical patients were analyzed across all three frailty indices.

**Table 1:** Association of patient demographics, comorbidities, and Risk Analysis Index (RAI) tiers

Variable	Total (*n* = 11, 965)	Not frail (RAI ≤ 20) *n* = 4,619	Prefrail (RAI 21–30) *n* = 7,065	Frail (RAI 31*–*40) *n* = 278	Severely frail (RAI ≥ 41) *n* = 3	*P*-value
Age (yr)	70.5 ± 8.8	62.2 ± 6.1	75.5 ± 5.6	80.2 ± 5.7	80.3 ± 4.5	< 0.001
Sex, male	0 (0.0%)	0 (0.0%)	0 (0.0%)	0 (0.0%)	0 (0.0%)	-
White	11,076 (92.6%)	4,148 (89.8%)	6,663 (94.3%)	263 (94.6%)	2 (66.7%)	< 0.001
Non-white/Unknown	889 (7.4%)	471 (10.2%)	402 (5.7%)	15 (5.4%)	1 (33.3%)	< 0.001
Body mass index (kg/m^2^)	31.6 ± 7.5	32.7 ± 8.0	31.0 ± 7.1	31.0 ± 8.5	29.1 ± 11.1	< 0.001
**Functional status**						
Independent	11,538 (97.2%)	4,579 (99.8%)	6,867 (98.1%)	91 (32.7%)	1 (33.3%)	< 0.001
Partially dependent	323 (2.7%)	8 (0.2%)	135 (1.9%)	180 (64.7%)	0 (0.0%)	< 0.001
Totally dependent	10 (0.1%)	0 (0.0%)	1 (0.0%)	7 (2.5%)	2 (66.7%)	< 0.001
**Diabetes mellitus**						
No diabetes	9,726 (81.3%)	3,811 (82.5%)	5,710 (80.8%)	203 (73.0%)	2 (66.7%)	< 0.001
Oral medication	1,560 (13.0%)	559 (12.1%)	954 (13.5%)	47 (16.9%)	0 (0.0%)	< 0.001
Insulin	679 (5.7%)	249 (5.4%)	401 (5.7%)	28 (10.1%)	1 (33.3%)	< 0.001
COPD	933 (7.8%)	273 (5.9%)	610 (8.6%)	50 (18.0%)	0 (0.0%)	< 0.001
CHF	87 (0.7%)	0 (0.0%)	50 (0.7%)	37 (13.3%)	0 (0.0%)	< 0.001
Current smoker	1,164 (9.7%)	754 (16.3%)	397 (5.6%)	13 (4.7%)	0 (0.0%)	< 0.001
Dyspnea at rest	994 (8.3%)	84 (1.8%)	794 (11.2%)	115 (41.4%)	1 (33.3%)	< 0.001
Hypertension	8,266 (69.1%)	2,727 (59.0%)	5,306 (75.1%)	230 (82.7%)	3 (100.0%)	< 0.001
Disseminated cancer	22 (0.2%)	0 (0.0%)	7 (0.1%)	14 (5.0%)	1 (33.3%)	< 0.001
Steroid use	708 (5.9%)	299 (6.5%)	387 (5.5%)	21 (7.6%)	1 (33.3%)	0.015
Weight loss	27 (0.2%)	1 (0.0%)	19 (0.3%)	6 (2.2%)	1 (33.3%)	< 0.001
**mFI-5**						
Not frail (mFI-5 = 0)	3,335 (27.9%)	1,772 (38.4%)	1,555 (22.0%)	8 (2.9%)	0 (0.0%)	< 0.001
Prefrail (mFI-5 = 1)	7,051 (58.9%)	2,429 (52.6%)	4,547 (64.4%)	75 (27.0%)	0 (0.0%)	< 0.001
Frail (mFI-5 = 2)	1,311 (11.0%)	361 (7.8%)	810 (11.5%)	137 (49.3%)	3 (100.0%)	< 0.001
Severely frail (mFI-5 ≥ 3)	268 (2.2%)	57 (1.2%)	153 (2.2%)	58 (20.9%)	0 (0.0%)	< 0.001
**GNRI**						
> 98	9,970 (83.3%)	3,993 (86.5%)	5,777 (81.8%)	198 (71.2%)	2 (66.7%)	< 0.001
92*–*98	1,558 (13.0%)	528 (11.4%)	977 (13.8%)	53 (19.1%)	0 (0.0%)	< 0.001
82*–*91	390 (3.3%)	95 (2.1%)	272 (3.9%)	22 (7.9%)	1 (33.3%)	< 0.001
< 82	47 (0.4%)	3 (0.1%)	39 (0.6%)	5 (1.8%)	0 (0.0%)	< 0.001
**ASA**						
I	94 (0.8%)	65 (1.4%)	29 (0.4%)	0 (0.0%)	0 (0.0%)	< 0.001
II	4,590 (38.4%)	2,090 (45.3%)	2,450 (34.7%)	50 (18.0%)	0 (0.0%)	< 0.001
III	6,906 (57.7%)	2,386 (51.7%)	4,329 (61.3%)	189 (68.0%)	2 (66.7%)	< 0.001
IV	375 (3.1%)	78 (1.7%)	257 (3.6%)	39 (14.0%)	1 (33.3%)	< 0.001
Length of stay (days)	1.9 ± 2.2	1.6 ± 2.2	2.0 ± 2.0	3.5 ± 3.5	3.3 ± 0.6	< 0.001
Operative time (min)	103.2 ± 42.9	106.0 ± 44.3	101.1 ± 41.6	108.7 ± 47.0	81.0 ± 19.3	< 0.001

COPD, Chronic Obstructive Pulmonary Disease; CHF, Congestive Heart Failure; mFI-5, Modified Frailty Index-5; RAI, Risk Analysis Index; GNRI, Geriatric Nutritional Risk Index; ASA, American Society of Anesthesiologists

### Primary outcome analysis

For non-home discharge, RAI demonstrated superior discriminative ability with an AUC of 0.784 (95% CI 0.772–0.796), significantly outperforming mFI-5 (AUC 0.601, 95% CI 0.587–0.615, *P* < 0.001) and GNRI (AUC 0.544, 95% CI 0.530–0.558, *P* < 0.001). The incidence of non-home discharge increased dramatically across RAI tiers: 2.4% in robust patients, 19.0% in prefrail patients, 48.6% in frail patients, and 66.7% in severely frail patients (*P* < 0.001). (Table 2) Extended length of stay analysis yielded similar findings, with RAI achieving an AUC of 0.670 (95% CI 0.658–0.682) compared to mFI-5 (AUC 0.590, 95% CI 0.577–0.603, *P* < 0.001) and GNRI (AUC 0.543, 95% CI 0.530–0.556, *P* < 0.001). The prevalence of extended stay demonstrated a clear gradient across RAI categories: 10.9% robust, 23.8% prefrail, 55.4% frail, and 100% severely frail (*P* < 0.001) (Table 3).

**Table 2:** 30-day outcome measures by RAI tiers

Variable	Total	Not frail (RAI ≤ 20) *n* = 4,619	Prefrail (RAI 21*–*30) *n* = 7,065	Frail (RAI 31*–*40) *n* = 278	Severely frail (RAI ≥ 41) *n* = 3	*P*-value
Mortality	19 (0.16%)	5 (0.11%)	13 (0.18%)	1 (0.36%)	0 (0.00%)	0.628
Non-home discharge	1,589 (13.3%)	110 (2.4%)	1,342 (19.0%)	135 (48.6%)	2 (66.7%)	< 0.001
Extended LOS	2,344 (19.6%)	504 (10.9%)	1,683 (23.8%)	154 (55.4%)	3 (100.0%)	< 0.001
Major complications	112 (0.94%)	38 (0.82%)	68 (0.96%)	6 (2.16%)	0 (0.00%)	0.158
Minor complications	103 (0.86%)	30 (0.65%)	63 (0.89%)	10 (3.60%)	0 (0.00%)	< 0.001
Readmission	373 (3.12%)	115 (2.49%)	236 (3.34%)	22 (7.91%)	0 (0.00%)	< 0.001
Reoperation	147 (1.23%)	65 (1.41%)	76 (1.08%)	6 (2.16%)	0 (0.00%)	0.204

LOS, Length of Stay

**Table 3:** 30-day outcome measures by Geriatric Nutritional Risk Index (GNRI) tiers

Variable	Total	GNRI > 98 *n* = 9,970	GNRI 92*–*98 *n* = 1,558	GNRI 82*–*91 *n* = 390	GNRI < 82 *n* = 47	*P*-value
Mortality	19 (0.16%)	12 (0.12%)	6 (0.39%)	0 (0.00%)	1 (2.13%)	< 0.001
Non-home discharge	1,589 (13.3%)	1,153 (11.57%)	285 (18.34%)	120 (30.77%)	31 (65.96%)	< 0.001
Extended LOS	2,344 (19.6%)	1,750 (17.55%)	410 (26.32%)	155 (39.74%)	29 (61.70%)	< 0.001
Major complications	112 (0.94%)	86 (0.86%)	17 (1.09%)	8 (2.05%)	1 (2.13%)	0.074
Minor complications	103 (0.86%)	75 (0.75%)	18 (1.16%)	9 (2.31%)	1 (2.13%)	0.004
Readmission	373 (3.12%)	284 (2.85%)	61 (3.92%)	23 (5.90%)	5 (10.64%)	< 0.001
Reoperation	147 (1.23%)	119 (1.19%)	20 (1.28%)	7 (1.79%)	1 (2.13%)	0.686

LOS, Length of Stay

### Secondary outcome analysis

Thirty-day mortality occurred in 19 patients (0.16%), with RAI showing modest discriminative ability (AUC 0.548, 95% CI 0.456–0.640), compared to mFI-5 (AUC 0.532, 95% CI 0.441–0.623) and GNRI (AUC 0.589, 95% CI 0.498–0.680). Major complications affected 112 patients (0.94%), with RAI achieving an AUC of 0.548 (95% CI 0.494–0.602) compared to mFI-5 (AUC 0.537, 95% CI 0.483–0.591) and GNRI (AUC 0.662, 95% CI 0.608–0.716). Minor complications occurred in 103 patients (0.86%), with significant variation across RAI tiers (0.65% robust, 0.89% prefrail, 3.60% frail, *P* < 0.001). RAI demonstrated an AUC of 0.595 (95% CI 0.541–0.649) for this outcome. Unplanned readmission within 30 days affected 373 patients (3.12%), with RAI showing moderate predictive ability (AUC 0.571, 95% CI 0.538–0.604) (Table 4).

**Table 4:** Univariate logistic regression analysis of GNRI and RAI for major postoperative outcomes

Outcome	GNRI category	Odds ratio (95% CI)	RAI category	Odds ratio (95% CI)
**Mortality**				
	92*–*98	-	21*–*30	1.64 (0.58*–*4.64)
	82*–*91	4.66 (0.56*–*38.9)	31*–*40	3.27 (0.39*–*27.5)
	< 82	25.5 (2.28*–*285)	≥ 41	-
**Non-home discharge**				
	92*–*98	2.70 (2.26*–*3.22)	21*–*30	9.59 (7.84*–*11.7)
	82*–*91	3.82 (3.04*–*4.80)	31*–*40	43.8 (33.7*–*57.0)
	< 82	16.2 (8.72*–*30.1)	≥ 41	66.7 (6.06*–*734)
**Extended LOS**				
	92*–*98	3.14 (2.65*–*3.72)	21*–*30	2.57 (2.29*–*2.88)
	82*–*91	1.68 (1.33*–*2.13)	31*–*40	10.9 (8.34*–*14.2)
	< 82	7.63 (4.26*–*13.7)	≥ 41	-
**Major complications**				
	92*–*98	2.41 (0.30*–*19.2)	21*–*30	1.17 (0.77*–*1.78)
	82*–*91	1.28 (0.76*–*2.16)	31*–*40	2.67 (1.12*–*6.35)
	< 82	2.50 (0.32*–*19.7)	≥ 41	-
**Minor complications**				
	92*–*98	3.14 (1.56*–*6.32)	21*–*30	1.38 (0.89*–*2.14)
	82*–*91	1.56 (0.93*–*2.62)	31*–*40	5.83 (2.90*–*11.7)
	< 82	2.90 (0.39*–*21.7)	≥ 41	-
**Readmission**				
	92*–*98	2.13 (1.36*–*3.34)	21*–*30	1.35 (1.08*–*1.70)
	82*–*91	1.40 (1.07*–*1.83)	31*–*40	3.43 (2.16*–*5.44)
	< 82	4.03 (1.55*–*10.5)	≥ 41	-
**Reoperation**				
	92*–*98	1.51 (0.68*–*3.36)	21*–*30	0.76 (0.55*–*1.05)
	82*–*91	1.08 (0.66*–*1.76)	31*–*40	1.54 (0.65*–*3.64)
	< 82	1.80 (0.24*–*13.3)	≥ 41	-

GNRI, Geriatric Nutritional Risk Index; RAI, Risk Analysis Index; CI, Confidence Interval; LOS, Length of Stay; Reference groups: GNRI > 98 and RAI ≤ 20

### Multivariable analysis

In multivariable logistic regression models incorporating all three frailty indices, RAI maintained independent associations with primary outcomes. For non-home discharge, each 10-point increase in RAI was associated with an odds ratio of 2.89 (95% CI 2.71–3.08, *P* < 0.001), while mFI-5 (OR 1.12, 95% CI 1.03–1.22, *P* = 0.008) and GNRI (OR 0.97, 95% CI 0.96–0.98, *P* < 0.001) showed weaker associations. Similar patterns emerged for extended length of stay, with RAI demonstrating the strongest independent association (OR per 10-point increase: 1.72, 95% CI 1.65–1.79, *P* < 0.001) (Table 5).

**Table 5:** Multivariable regression analysis of major complications

Variable	Adjusted OR	95% CI Lower	95% CI Upper	*P*-value
ASA	1.35	0.91	1.99	0.136
GNRI	0.99	0.95	1.03	0.610
RAI	1.02	0.97	1.07	0.468

OR, Odds Ratio; CI, Confidence Interval; ASA, American Society of Anesthesiologists; GNRI, Geriatric Nutritional Risk Index; RAI, Risk Analysis Index

### Internal validation

Bootstrap validation with 100 replications confirmed the stability of AUC estimates. For non-home discharge, RAI maintained a bias-corrected AUC of 0.778 (95% CI 0.766–0.790), while mFI-5 and GNRI showed bias-corrected AUCs of 0.596 (95% CI 0.582–0.610) and 0.539 (95% CI 0.525–0.553), respectively. Extended length of stay validation yielded similar results, with RAI achieving a bias-corrected AUC of 0.663 (95% CI 0.651–0.675) (Tables 6 and 7).

**Table 6:** Area Under the Curve (AUC) analysis for frailty indices and postoperative outcomes

Outcome Variable	Index	AUC	95% CI Lower	95% CI Upper	*P*-value*
**Major Complications**					
	RAI	0.548	0.494	0.591	Ref
	ASA	0.592	0.541	0.630	0.044
	GNRI	0.662	0.602	0.708	< 0.001
	mFI-5	0.537	0.484	0.580	0.689
**Minor Complications**					
	RAI	0.595	0.529	0.648	Ref
	ASA	0.624	0.574	0.661	0.334
	GNRI	0.584	0.533	0.623	0.712
	mFI-5	0.578	0.519	0.626	0.635
**Non-home discharge**					
	RAI	0.784	0.772	0.796	Ref
	ASA	0.622	0.608	0.636	< 0.001
	GNRI	0.544	0.530	0.558	< 0.001
	mFI-5	0.601	0.587	0.615	< 0.001
**Extended LOS**					
	RAI	0.670	0.658	0.682	Ref
	ASA	0.610	0.598	0.622	< 0.001
	GNRI	0.543	0.530	0.556	< 0.001
	mFI-5	0.590	0.577	0.603	< 0.001
**Readmission**					
	RAI	0.571	0.538	0.604	Ref
	ASA	0.601	0.569	0.633	0.175
	GNRI	0.582	0.549	0.615	0.601
	mFI-5	0.577	0.544	0.610	0.805
**Reoperation**					
	RAI	0.528	0.483	0.573	Ref
	ASA	0.559	0.515	0.603	0.207
	GNRI	0.539	0.494	0.584	0.648
	mFI-5	0.556	0.511	0.601	0.312

^*^P-value from DeLong test comparing each index to RAI

AUC, Area Under the Curve; CI, Confidence Interval; RAI, Risk Analysis Index; ASA, American Society of Anesthesiologists; GNRI, Geriatric Nutritional Risk Index; mFI-5, Modified Frailty Index-5; LOS, Length of Stay

**Table 7:** Internal validation of AUC analysis by bootstrap resampling

Outcome Variable	Index	Initial AUC	Validated AUC	Bias-Corrected CI Lower	Bias-Corrected CI Upper
**Major Complications**					
	RAI	0.548	0.542	0.494	0.591
	ASA	0.592	0.586	0.541	0.630
	GNRI	0.662	0.655	0.602	0.708
	mFI-5	0.537	0.532	0.484	0.580
**Minor Complications**					
	RAI	0.595	0.589	0.529	0.648
	ASA	0.624	0.618	0.574	0.661
	GNRI	0.584	0.578	0.533	0.623
	mFI-5	0.578	0.573	0.519	0.626
**Non-home discharge**					
	RAI	0.784	0.778	0.766	0.790
	ASA	0.622	0.616	0.602	0.630
	GNRI	0.544	0.539	0.525	0.553
	mFI-5	0.601	0.596	0.582	0.610
**Extended LOS**					
	RAI	0.670	0.663	0.651	0.675
	ASA	0.610	0.604	0.592	0.616
	GNRI	0.543	0.538	0.525	0.551
	mFI-5	0.590	0.584	0.571	0.597
**Readmission**					
	RAI	0.571	0.565	0.532	0.598
	ASA	0.601	0.595	0.562	0.628
	GNRI	0.582	0.576	0.543	0.609
	mFI-5	0.577	0.571	0.538	0.604
**Reoperation**					
	RAI	0.528	0.523	0.477	0.569
	ASA	0.559	0.554	0.509	0.599
	GNRI	0.539	0.534	0.488	0.580
	mFI-5	0.556	0.551	0.505	0.597

AUC, Area Under the Curve; CI, Confidence Interval; RAI, Risk Analysis Index; ASA, American Society of Anesthesiologists; GNRI, Geriatric Nutritional Risk Index; mFI-5, Modified Frailty Index-5; LOS, Length of Stay

## Discussion

### Principal findings and clinical significance

This large-scale comparative analysis demonstrates that the Risk Analysis Index provides superior predictive performance for critical postoperative outcomes compared to traditional frailty measures in elderly female patients undergoing total shoulder arthroplasty. The RAI’s strong discrimination for non-home discharge (AUC 0.784) and extended length of stay (AUC 0.670) represents a substantial improvement over the mFI-5 and GNRI, both of which showed limited predictive utility with AUC values approaching 0.5 for several outcomes.

The clinical implications of these findings are substantial. Non-home discharge and extended length of stay represent not only important quality metrics but also surrogate markers for functional decline, increased healthcare costs, and patient dissatisfaction. Panayi et al. [16] demonstrated that loss of functional independence after surgery in older patients was associated with increased complications and adverse discharge destinations. Similarly, Mekkawy et al. [17] found that factors predicting non-home discharge after orthopedic procedures included both frailty metrics and functional status indicators. The RAI’s ability to identify patients at highest risk for these outcomes—with nearly half of frail patients experiencing non-home discharge—enables targeted preoperative optimization and discharge planning interventions. Importantly, the RAI should not be interpreted as a universal predictor of all adverse events. Its performance for 30-day mortality (AUC 0.548) and major complications (AUC 0.548) approached chance levels, indicating poor discriminative ability for these outcomes. The RAI’s clinical utility in this population is specifically concentrated in predicting discharge disposition and length of stay rather than medical complications or death.

### Comparison with existing literature

Our results align with and extend previous investigations of frailty assessment in orthopedic surgery. The declining performance of mFI-5 observed in our cohort corroborates concerns raised by Gani et al. [8] regarding missing data in NSQIP affecting mFI calculation, with 100% of data missing for five of eleven variables after 2012. Shultz et al. [9] similarly demonstrated that systematic changes in the NSQIP database resulted in missing data for many variables included in the mFI, potentially affecting studies utilizing this index. Aziz et al. [10] further emphasized that filtering patient cohorts according to missing data significantly affected analyses of predictors for major complications.

The RAI’s superior performance can be attributed to its multidimensional assessment framework, which captures not only comorbidity burden but also functional status and recent physiological decline. Desai et al. [11] demonstrated the value of incorporating functional status in predicting outcomes after upper extremity procedures, finding that the 5-mFI, including functional status, was significantly associated with complications. This comprehensive approach appears particularly relevant in shoulder arthroplasty, where postoperative functional recovery depends heavily on baseline reserve and rehabilitation potential.

Previous studies have validated the importance of functional metrics in orthopedic risk assessment. Leven et al. [5] showed that increasing frailty scores were associated with increased complications and prolonged length of stay in spine surgery patients. Traven et al. [4] demonstrated that the mFI-5 was a strong predictor for complications and extended hospital stay in geriatric hip fracture patients, with risk increasing by 29.8% for each point increase. Our findings extend these observations to the shoulder arthroplasty population.

The limited utility of GNRI in our cohort contrasts with findings in trauma populations but aligns with recent shoulder arthroplasty studies. Malik et al. [6] found GNRI predictive in hip fracture patients, while Phen et al. [12] demonstrated that combining frailty and nutritional assessment improved risk prediction. However, in elective shoulder arthroplasty, isolated nutritional parameters appear inadequate for comprehensive risk assessment. This discrepancy may reflect differences between acute trauma and elective surgical settings.

### Mechanistic insights and biological plausibility

The RAI’s incorporation of functional status likely explains much of its predictive superiority. Functional dependence represents a final common pathway of multiple age-related processes, including sarcopenia, neurodegeneration, and cardiopulmonary decline. Zhang et al. [18] found that frailty measures incorporating functional assessment better predicted the range of motion and reoperation after reverse TSA for fracture. In shoulder arthroplasty, where postoperative rehabilitation demands significant physical and cognitive engagement, baseline functional limitations may profoundly impact recovery trajectories.

Furthermore, the RAI’s inclusion of recent weight loss captures dynamic physiological changes that static comorbidity counts miss. Unintentional weight loss often reflects underlying inflammatory states, occult malignancy, or accelerated catabolism—all of which compromise surgical stress responses and wound healing. Sarkar et al. [19] demonstrated that frailty indices incorporating dynamic markers better predicted ambulation loss after vascular procedures. This dynamic assessment proves particularly valuable in elderly populations where subclinical disease processes frequently precede overt clinical manifestations.

### Implementation considerations

The practical advantages of RAI implementation merit emphasis. All component variables are routinely collected during preoperative assessment, requiring no additional testing or specialized equipment. Unlike the mFI, which suffers from systematic missing data issues in NSQIP as highlighted by Gani et al. [8] and Shultz et al. [9], the RAI relies on consistently reported variables. The straightforward calculation algorithm facilitates real-time risk stratification during surgical consultation. Healthcare systems could leverage RAI stratification to develop tiered perioperative pathways. Gonzalez et al. [20] demonstrated that frailty-guided protocols reduced complications in lower extremity reconstruction. Similarly, Andersen et al. [21] showed that targeted interventions based on comprehensive frailty assessment improved outcomes in orthopedic patients. Such risk-based resource allocation could improve outcomes while maintaining operational efficiency.

### Study strengths and limitations

This investigation’s strengths include its large sample size, multi-institutional data source, and comprehensive comparative framework. The focus on elderly female patients addresses an important but understudied population in orthopedic risk modeling. The rigorous statistical methodology, including bootstrap validation and multivariable adjustment, enhances confidence in our findings.

However, important limitations warrant consideration. The retrospective design precludes causal inference, and NSQIP’s 30-day follow-up period may miss delayed complications or functional outcomes. As noted by Lemos et al. [22], the majority of frailty studies in orthopedics focus on short-term complications rather than functional recovery or patient-reported outcomes. The database lacks patient-reported outcomes, limiting the assessment of quality of life impacts. Additionally, unmeasured confounders such as social support, cognitive function, and rehabilitation access may influence outcomes independently of measured frailty.

The exclusion of patients with missing data, while necessary for analysis integrity, may introduce selection bias if missingness patterns correlate with frailty or outcomes. As Aziz et al. [10] demonstrated, the mismanagement of missing data can lead to inaccurate findings. Additionally, the studied population was relatively healthy, with 97.7% classified as robust or prefrail by RAI, and only 281 patients (2.3%) classified as frail or severely frail. This restricted spectrum of frailty may have attenuated the observed predictive power of all indices and limits generalizability to populations with higher frailty burden. Finally, external validation in prospective cohorts remains necessary to confirm the RAI’s superiority and establish optimal risk thresholds for clinical interventions. The absence of a pre-defined risk standard means our analysis focused on comparative discrimination rather than absolute risk calibration; establishing validated RAI cut points for clinical decision-making will require prospective studies with pre-specified intervention thresholds.

### Future directions

Several avenues warrant further investigation. Prospective validation studies should assess whether RAI-guided interventions improve outcomes in high-risk patients. Vankara et al. [21] recently showed that newer frailty indices like the Pathologic Fracture Mortality Index (PFMI) outperformed traditional measures, suggesting continued evolution in frailty assessment is needed. Integration with emerging biomarkers of aging might further enhance predictive accuracy.

Additionally, investigation of sex-specific frailty patterns remains crucial. While this study focused on female patients, comparative analyses in male cohorts could identify important biological or social modifiers of frailty-outcome relationships. Gupta et al. [22] emphasized the heterogeneity in frailty measurement across orthopedic subspecialties, highlighting the need for procedure-specific validation. Finally, economic analyses quantifying the cost-effectiveness of RAI implementation would support broader adoption in value-based care models.

## Conclusion

The Risk Analysis Index demonstrates superior predictive performance compared to traditional frailty measures for non-home discharge and extended length of stay in elderly female patients undergoing total shoulder arthroplasty, with its strongest discrimination observed for discharge disposition (AUC 0.784). Its strong discrimination for non-home discharge (AUC 0.784) represents the primary advantage over mFI-5 and GNRI, which showed AUC values near 0.5–0.6 for most outcomes; this, combined with practical implementation advantages and avoidance of missing data issues that affect mFI-based tools, supports adoption of RAI as the preferred tool for discharge planning in this population. By enabling accurate preoperative risk assessment, the RAI facilitates personalized surgical planning and targeted interventions to optimize outcomes in an increasingly frail surgical population. Future research should focus on prospective validation and development of RAI-guided intervention protocols to translate improved risk prediction into enhanced clinical outcomes.

## Data Availability

The data used in this study are available from the American College of Surgeons National Surgical Quality Improvement Program. Access requires application approval through the ACS NSQIP Participant Use Data File program.

## References

[1] Wagner ER, Farley KX, Higgins I, Wilson JM, Daly CA, Gottschalk MB. The incidence of shoulder arthroplasty: rise and future projections compared with hip and knee arthroplasty. J Shoulder Elbow Surg. 2020;29:2601–9. 10.1016/j.jse.2020.03.049. 33190759 10.1016/j.jse.2020.03.049

[2] Holzgrefe RE, Wilson JM, Staley CA, Anderson TL, Wagner ER, Gottschalk MB. Modified frailty index is an effective risk-stratification tool for patients undergoing total shoulder arthroplasty. J Shoulder Elbow Surg. 2019;28:1232–40. 10.1016/j.jse.2018.12.004. 30878278 10.1016/j.jse.2018.12.004

[3] Ling K, Achonu JU, Martino R, Liu SH, Komatsu DE, Wang ED. Six-item modified frailty index independently predicts complications following total shoulder arthroplasty. JSES Int. 2024;8:99–103. 10.1016/j.jseint.2023.08.010. 38312266 10.1016/j.jseint.2023.08.010 PMC10837715

[4] Traven SA, Reeves RA, Althoff AD, Slone HS, Walton ZJ. New five-factor modified frailty index predicts morbidity and mortality in geriatric hip fractures. J Orthop Trauma. 2019;33:319–23. 10.1097/BOT.0000000000001455. 30730361 10.1097/BOT.0000000000001455

[5] Leven DM, Lee NJ, Kim JS, Kothari P, Steinberger J, Guzman J, et al. Frailty is predictive of adverse postoperative events in patients undergoing lumbar fusion. Glob Spine J. 2017;7:529–35. 10.1177/2192568217700099. 10.1177/2192568217700099 PMC5582713 28894682

[6] Malik AT, Quatman-Yates C, Phieffer LS, Ly TV, Khan SN, Quatman CE. Factors associated with inability to bear weight following hip fracture surgery: an analysis of the ACS-NSQIP hip fracture procedure targeted database. Geriatr Orthop Surg Rehabil. 2019. 10.1177/2151459319837481. 31069126 10.1177/2151459319837481 PMC6492357

[7] Bzovsky S, Comeau-Gauthier M, Schemitsch EH, Swiontkowski M, Heels-Ansdell D, Frihagen F, et al. Factors associated with mortality after surgical management of femoral neck fractures. J Orthop Trauma. 2020;34:S15-21. 10.1097/BOT.0000000000001937. 33027161 10.1097/BOT.0000000000001937

[8] Gani F, Canner JK, Pawlik TM. Use of the modified frailty index in the American College of Surgeons National Surgical Improvement Program database. JAMA Surg. 2017;152:205. 10.1001/jamasurg.2016.3479. 27784060 10.1001/jamasurg.2016.3479

[9] Shultz BN, Ottesen TD, Ondeck NT, Bovonratwet P, McLynn RP, Cui JJ, et al. Systematic changes in the National Surgical Quality Improvement Program database over the years can affect comorbidity indices such as the Modified Frailty Index and Modified Charlson Comorbidity Index for lumbar fusion studies. Spine (Phila Pa 1976). 2018;43:798–804. 10.1097/BRS.0000000000002418. 28922281 10.1097/BRS.0000000000002418

[10] Aziz KT, Nayar SK, LaPorte DM, Ingari JV, Giladi AM. Impact of missing data on identifying risk factors for postoperative complications in hand surgery. Hand. 2022;17:1257–63. 10.1177/15589447211023867. 34154440 10.1177/15589447211023867 PMC9608303

[11] Desai A, Luo A, Borowsky PA, Bustos VP, Fullerton N, Xu KY, et al. Evaluation of modified frailty index for predicting postoperative outcomes after upper extremity replantation and revascularization procedures. J Reconstr Microsurg. 2025;41:557–65. 10.1055/a-2460-4706. 39496316 10.1055/a-2460-4706

[12] Phen HM, Jones C, Kravets VG, Farley KX, Schwartz AM, Wilson JM, et al. Impact of frailty and malnutrition on outcomes after surgical fixation of lower extremity fractures in young patients. J Orthop Trauma. 2021;35:e126–33. 10.1097/BOT.0000000000001952. 32910628 10.1097/BOT.0000000000001952

[13] Hall DE, Arya S, Schmid KK, Blaser C, Carlson MA, Bailey TL, et al. Development and initial validation of the risk analysis index for measuring frailty in surgical populations. JAMA Surg. 2017;152:175. 10.1001/jamasurg.2016.4202. 27893030 10.1001/jamasurg.2016.4202 PMC7140150

[14] Panayi AC, Knoedler S, Didzun O, Ghanad I, Kneser U, Hundeshagen G, et al. Loss of functional independence after plastic surgery in older patients: American College of Surgeons National Surgical Quality Improvement Program database. Plast Reconstr Surg Glob Open. 2024;12:e6167. 10.1097/GOX.0000000000006167. 39267727 10.1097/GOX.0000000000006167 PMC11392476

[15] Mekkawy KL, Chaudhry YP, Rao SS, Barry K, Puvanesarajah V, Amin RM, et al. Predictors of hospice discharge after surgical fixation of hip fractures. J Am Acad Orthop Surg. 2023;31:e35-43. 10.5435/JAAOS-D-21-01015. 36394941 10.5435/JAAOS-D-21-01015

[16] Zhang D, Ostergaard PJ, Hall MJ, Shoji M, Earp BE. The relationship between frailty and functional outcomes, range of motion, and reoperation after reverse total shoulder arthroplasty for proximal humerus fracture. Orthopedics. 2023;46:274–9. 10.3928/01477447-20230330-02. 37018624 10.3928/01477447-20230330-02

[17] Sarkar A, St. John A, Nagarsheth KH. Predictive effect of frailty on amputation, mortality, and ambulation in patients undergoing revascularization for acute limb ischemia. Ann Vasc Surg. 2021;73:273–9. 10.1016/j.avsg.2020.10.048. 33340668 10.1016/j.avsg.2020.10.048

[18] Gonzalez M, Zietowski M, Patel R, Chattha A, Cripps CN, Beederman M. Applying the modified five-item frailty index to predict complications following lower extremity free flap reconstruction in trauma patients. J Reconstr Microsurg. 2025. 10.1055/a-2508-6716. 39875120 10.1055/a-2508-6716

[19] Andersen JC, Gabel JA, Mannoia KA, Kiang SC, Patel ST, Teruya TH, et al. 5-item modified frailty index predicts outcomes after below-knee amputation in the Vascular Quality Initiative amputation registry. Am Surg. 2020;86:1225–9. 10.1177/0003134820964190. 33106001 10.1177/0003134820964190

[20] Lemos JL, Welch JM, Xiao M, Shapiro LM, Adeli E, Kamal RN. Is Frailty Associated with Adverse Outcomes After Orthopaedic Surgery? JBJS Rev 2021;9. 10.2106/JBJS.RVW.21.00065. 10.2106/JBJS.RVW.21.00065 34936580

[21] Vankara A, Leland CR, Maxson R, Raad M, Sabharwal S, Morris CD, et al. Predicting Risk of 30-day Postoperative Morbidity Using the Pathologic Fracture Mortality Index. J Am Acad Orthop Surg. 2024;32:e146–55. 10.5435/JAAOS-D-23-00297. 37793148 10.5435/JAAOS-D-23-00297

[22] Gupta NK, Dunivin F, Chmait HR, Smitterberg C, Buttar A, Fazal-ur-Rehman M, et al. Orthopedic frailty risk stratification (OFRS): a systematic review of the frailty indices predicting adverse outcomes in orthopedics. J Orthop Surg Res. 2025;20:247. 10.1186/s13018-025-05609-2. 40051013 10.1186/s13018-025-05609-2 PMC11887260

